# Vitamin B12 among Vegetarians: Status, Assessment and Supplementation

**DOI:** 10.3390/nu8120767

**Published:** 2016-11-29

**Authors:** Gianluca Rizzo, Antonio Simone Laganà, Agnese Maria Chiara Rapisarda, Gioacchina Maria Grazia La Ferrera, Massimo Buscema, Paola Rossetti, Angela Nigro, Vincenzo Muscia, Gaetano Valenti, Fabrizio Sapia, Giuseppe Sarpietro, Micol Zigarelli, Salvatore Giovanni Vitale

**Affiliations:** 1 Vico Sant’andrea 5, Ritiro, Messina 98152, Italy; gianlucarizzo@email.it; 2 Unit of Gynecology and Obstetrics, Department of Human Pathology in Adulthood and Childhood, “G. Barresi”, University of Messina, Via Consolare Valeria 1, Messina 98125, Italy; vitalesalvatore@hotmail.com; 3 Department of General Surgery and Medical Surgical Specialties, University of Catania, Via S. Sofia 78, Catania 95124, Italy; agni.rap@live.it (A.M.C.R.); valentigaetano@gmail.com (G.V.); fabrizio.sapia@libero.it (F.S.); peppe_sarpietro@hotmail.com (G.S.); micolziga@gmail.com (M.Z.); 4 Department of Gastroenterology and Digestive Endoscopy Maddalena Raimondi San Cataldo, Via Forlanini 5, San Cataldo, Caltanissetta 93017, Italy; giolaferrera@libero.it; 5 Unit of Diabetology and Endocrino-Metabolic Diseases, Hospital for Emergency Cannizzaro, Via Messina 829, Catania 95126, Italy; buscemamassimo@gmail.com (M.B.); paolarossetti@alice.it (P.R.); ang.nigro@gmail.com (A.N.); vince80@alice.it (V.M.)

**Keywords:** cobalamin, vitamin B12, vegetarian, vegan, food sources, deficiency, cardiovascular disease, neurological symptoms, supplements, diagnostic markers

## Abstract

Cobalamin is an essential molecule for humans. It acts as a cofactor in one-carbon transfers through methylation and molecular rearrangement. These functions take place in fatty acid, amino acid and nucleic acid metabolic pathways. The deficiency of vitamin B12 is clinically manifested in the blood and nervous system where the cobalamin plays a key role in cell replication and in fatty acid metabolism. Hypovitaminosis arises from inadequate absorption, from genetic defects that alter transport through the body, or from inadequate intake as a result of diet. With the growing adoption of vegetarian eating styles in Western countries, there is growing focus on whether diets that exclude animal foods are adequate. Since food availability in these countries is not a problem, and therefore plant foods are sufficiently adequate, the most delicate issue remains the contribution of cobalamin, which is poorly represented in plants. In this review, we will discuss the status of vitamin B12 among vegetarians, the diagnostic markers for the detection of cobalamin deficiency and appropriate sources for sufficient intake, through the description of the features and functions of vitamin B12 and its absorption mechanism.

## 1. Introduction

Recently, the vegetarian eating style has increased in popularity, with 10% of the population opting to exclude animal foods from their diet [1]. The decision to undertake such a choice depends primarily on ethical and ecological aspects, but also on health reasons. However, when the reasons are ethical, this may result in a reduced interest in the knowledge of the nutritional aspects of such a choice [2]. The scientific literature shows that the reduction or exclusion of animal foods may reduce the risk of Coronary Heart Disease (CHD) and Type 2 Diabetes (T2D) through modifiable factors such as body mass, serum glucose, blood pressure and serum lipid profile. These disorders contribute to a high mortality rate in Western countries [3,4,5,6,7]. Nevertheless, the risk of possible nutritional deficiencies in a non-balanced vegetarian diet, due to the absence of nutrients that can nullify these health benefits [8], should not be underestimated. In the European Prospective Investigation into Cancer and Nutrition-Oxford (EPIC-Oxford) cohort study, all-cause mortality among vegetarians in comparison with non-vegetarians was not significantly different [9]. In five prospective study analyses of 24,000 vegetarians among a total of 76,000 men and women, mortality from ischemic heart disease was lower in lacto-ovo-vegetarians than in vegans [10].

In the 2010 American Dietary Guidelines, the United States Department of Agriculture (USDA) proposed vegetarian adaptation patterns, including proposals for a balanced vegan diet [11]. Since 1988, and recently with the position paper of 2009, the American Dietetic Association has established that vegetarian diets are sustainable and safe for all age groups and in all physiological conditions, from childhood to old age, in athletes, and during pregnancy and lactation [12,13]. Sustainability relates to vegetarian diets that do not exclude dairy and eggs, as well as a vegan diet. However, it should be focused on proper planning and supplementation.

Vitamin B12, also called cobalamin (Cbl), is a water-soluble vitamin found in substantial quantities only in animal foods. If the consumption of animal foods is very low or absent, its scarce presence in plant foods makes its introduction essential, either through supplements or fortified foods. This deficiency is common among vegetarians and is the result of a very low intake [14]. Lower Cbl blood concentration can promote hematological shortages, resulting in increased mean corpuscular red cell volume (MCV) and anemia through the alteration of erythropoiesis [15]. Cbl also plays a key role in neuronal health, and a severe deficiency would inhibit the physiological formation of the myelin sheath, altering correct nerve transmission [15]. The slowing down of the degradation pathway of odd-chain fatty acids leads to an unusual incorporation of large quantities of C15 and C17 fatty acids in the nerve sheaths. This is due to an alteration of glial cell synthesis resulting in myelin modification [16].

There is no shared diagnostic consensus for Cbl deficiency, and blood Cbl cutoffs vary from one group to another. In addition, the concentration of Cbl in the blood may not be sensitive enough to detect early signs of a deficiency and should be accompanied by other markers in order to reach a correct diagnosis.

## 2. Background

Among vegetarians, there are differing styles that are categorized according to the level of animal food exclusion. Vegetarianism can be classified into different plant-based subgroups: lacto-ovo-vegetarian (LOV) which excludes animal flesh but includes eggs and dairy products; ovo-vegetarian (OV), similar to LOV but excluding dairy products; lacto-vegetarian (LV), similar to LOV but excluding eggs; or vegan (VN), which excludes all animal foods, dairy products and eggs. In common language, the term vegetarian is interpreted differently depending on the country (i.e., LOV in Italy or LV in India).

Unbalanced vegetarian diets could be lacking in nutrients that are poorly represented in vegetal foodstuffs or with a low bioavailability (e.g., iron, zinc, vitamin D, ω3 polyunsaturated fatty acids) [17]. However, only Cbl seems to be virtually absent in vegetables and its shortage can have serious implications.

A common mistake is to think that the presence of dairy products and eggs in the diet, as in LOV, can still ensure a proper intake of Cbl, despite excluding animal flesh. In reality, consumption of such foods, despite containing significant amounts of Cbl, would be sufficient neither on a daily basis nor in order to meet vitamin requirements [18]. A Dietary Reference Intake (DRI) of 2.4 μg/day for Cbl in adults is a common chosen value [19,20]. Such an amount is apparently exceeded by American adults, with a mean intake ranging from 4.6 to 6.3 μg/day [21]. However, it is not uncommon to see a moderate deficiency among omnivores in Western countries [22,23]. A recent report by the European Food Safety Authority (EFSA) Panel on Dietetic Products, Nutrition and Allergies established an Adequate Intake (AI) of 4 μg/day for adults, with a mean intake in European countries ranging between 4.2 and 8.6 μg/day [24].

The main source of consumption in the general population comes from animal foods with a significant contribution from milk and dairy products [25]. Losses of up to 50% can occur through food processing which involves cooking, pasteurization and exposure to fluorescent light. This limits its availability, together with a drop in absorption capacity and an increase in Cbl concentration in food [26]. Some researchers claim that the currently recommended intake levels may not be sufficient for an adequate daily intake, with particular regard to aging and the physiological reduction in absorptive capacity [27]. With senescence, the epithelial cells of the stomach reduce their ability to biosynthesize the transporter proteins of Cbl. The gastric secretion ability is necessary both for the dissociation of Cbl from foods and for the binding to the carriers [28]. For these reasons, the American Institute of Medicine recommends a supplementation of Cbl for people of 50 years of age and older [19]. The development of blood and cognitive disorders are rather common aspects found among the elderly population [29].

In the vegetarian diet, there are few sources of Cbl, whilst supplement use is frequently resisted.

Although some plant foods seem to represent a significant source of Cbl [30,31], data in the literature are still insufficient to determine whether Cbl is found in the active form, and whether regular consumption of these foods can be sustainable when the variability in the production processes is taken into account.

## 3. Chemical Properties of Cobalamin and Vitamin Activity

The synthesis of Cbl is a prerogative of some bacteria and archaea [32]. Its presence in animal foodstuffs depends on the process of biomagnification through food chains [30]. Small quantities found in plants and derived from biofilms bound by Cbl cannot be synthesized by either animals or plants [33]. Cbl is the vitamin with the largest steric hindrance (1355.4 Da) [34]. It consists of a tetrapyrrolic corrin ring core with a central cobalt atom, grouped with four nitrogen, one nucleotide base group and one upper ligand. The cobalt atom accepts different ligands on the upper surface: hydroxyl (Hydroxycobalamin-*H*-Cbl), deoxy-5′-adenosine (Deoxy-5′-adenosylcobalamin-Ado-Cbl), metyl (methylcobalamin-Me-Cbl), cyanide (Cyanocobalamin Cn-Cbl). Me-Cbl and Ado-Cbl are the active forms of vitamins used as coenzymes in the cell [35,36]. On the lower surface of the ring, the cobalt atom binds a 5,6-dimethylimidazole nucleotide base (DMB). Naturally occurring corrinoids without vitamin activity bind lower ligands different from DMB such as 2-methyladenylcobamide, methylmercaptoadenylcobamide-2,5-methoxybenzimidazolylcobamide, benzimidazolylcobamide, 5-hydroxybenzimidazolylcobamide, *p*-cresolylcobamide [30].

Me-Cbl is the cofactor of the enzyme methionine synthase participating in the metabolic homocysteine pathway (HCY), which is processed into methionine with the involvement of vitamin B6 and folate [37]. The reaction takes place in the cytosolic environment and the deficiency of vitamin cofactors leads to an increase in HCY blood concentration [38]. This pathway is critical in the regeneration of the methyl donor *S*-adenosylmethionine (SAM) and its dysfunction creates a shortage, affecting DNA synthesis and the physiological processes that require intense cell replication, such as hematopoietic process of the erythrocytes. Ado-Cbl is the vitamin cofactor of the methylmalonyl-CoA mutase enzyme. It works within the mitochondria, in the metabolism of branched-chain amino acids and fatty acid with an odd number of carbon atoms. These atoms are not subject to degradation by a beta-oxidation pathway accepting only two carbon units [39,40]. The shortage of Ado-Cbl leads to an accumulation of methylmalonic acid (MMA), an intermediate molecule of this pathway. A deficiency of this vitamin form is due primarily to neurological effects. The myelin sheaths of neurons are highly dependent on fatty acid metabolism and the low bioavailability of Ado-Cbl in neurons leads to the depletion of the myelin layer with dysfunctional nerve transmission. The role of Cbl in neuropathological lesions seems to be due to interactions with neurotrophic molecules such as Myelinolytic Tumor Necrosis Factor α (TNF-α), Epidermal Grow Factor (EGF) and Interleukin-6 (IL6) [41]. HCY itself appears to show a neurotoxic effect on synaptic receptors [42].

Cn-Cbl and H-Cbl are provitamin forms that require activation in cofactors Me-Cbl or Ado-Cbl to be utilized by the cells. Cn-Cbl is the form of Cbl first isolated and, although an artifact, it was initially identified as an anti-pernicious anemia factor [43]. Cobalamin performs a secondary function, that of removing potentially harmful molecules from the body. As a result, a small amount of Cbl in the blood stream is present in the Cn-Cbl form that binds cyanide residues. Cyanide is normally present in trace amounts in foods such as cassava, bitter almonds and apricot kernels (responsible for the bitter taste) [44]. H-Cbl is a form of cobalamin highly represented as a physiological intermediate [35]. Other corrinoids are non-vitamin analogs which are capable of engaging the carriers. These become less available to bind the vitamin forms, with an overall antinutrient outcome [45].

In the cell, the Cbl isoforms are metabolized inside the peroxisome by the reactions of dealkylation, decyanation and reduction, and then released in the specific cell compartment as coenzyme Me-Cbl and Ado-Cbl, according to their cytosolic or mitochondrial fate, respectively [46]. This step is crucial in the activation of the provitamin forms. Other corrinoid compounds do not fulfill the vitamin functions, in all probability due to the binding power of the lower ligand with the cobalt, which does not allow peroxisome activation [45]. It seems that all isoforms, provitamins and coenzymes should follow a mandatory route before being assigned to the appropriate cell district. However, the H-Cbl form may be more reactive, and its use can be facilitated by a number of enzymatic processes through non-specific cellular reactions [47]. If this mechanism were confirmed, the use of already active Cbl cofactors would not represent any provitamin advantage [45]. In 1982, Gimsing et al. analyzed Cbl in tissues from patients with pernicious anemia. After administration of H-Cbl or Cn-Cbl, they found that detected plasma Cbl was dependent on the form administered, dominant in the blood pattern. Results from erythrocytes and liver biopsies showed no differences, irrespective of the Cbl form used, indicating that administered Cbl preparations are converted in vivo to the necessary coenzymes [48]. Although current studies have many limitations and altogether there are no significant differences, the retention percentage after oral ingestion may change between different forms [49]. Following the ingestion of 1 μg of Cbl, the retention of Ado-Cbl and H-Cbl is 34% and 56%, respectively. Following the ingestion of 5 μg of Cbl, the retention of Ado-Cbl and Cn-Cbl is 13% and 20%, respectively. Following the ingestion of 25 μg of Cbl, the retention of Cn-Cbl and Ado-Cbl is 6% and 8%, respectively [50]. Updated data are not currently available.

## 4. Absorption and Transport

The absorption and transport of Cbl occurs via a complex network of proteins, which perform the task of optimizing the management of Cbl through the body districts. The study of rare genetic defects in carriers provided an opportunity to improve our understanding of the transport system and cellular trafficking of cobalamins [47,51]. Cbl is primarily associated with foodstuff proteins, such as Me-Cbl and Ado-Cbl. Free form, called crystalline or protein-unbound, is less represented and derived predominantly from supplements and fortified foods [25]. Transporters accept provitamin and coenzyme forms indifferently [52,53]. Following ingestion, the Cbl binds to the first salivary carrier R-binder or transcobalamin I, belonging to the corrin family of proteins. Its binding is non-specific and it binds various types of corrinoids, including antinutrient forms [52]. The increase of salivation promotes the secretion of R-binder and so the binding to the carrier that will occur after the dissociation from food proteins [54]. The gastric epithelial cells secrete hydrochloric acid and pepsin, responsible for the dissociation of Cbl from food proteins that enables binding to R-binder. The parietal cells also produce intrinsic factor (IF), another much more specific transporter for vitamin forms that binds Cbl released into the duodenum after cleavage from the R-binder through the use of trypsin and other pancreatic enzymes [55]. Thanks to the differing binding specificity, inactive analogs remain linked to R-binder and are excreted, while the vitamin forms continue on the route of absorption [56]. Binding to IF is critical for absorption into the terminal ileum, which is brought about by the membrane protein cubilin as part of the cubam receptor complex along with an amnion-less protein. The IF-Cbl complex is internalized by receptor-mediated endocytosis on the luminal side of the polarized enterocyte [57]. Inside the enterocyte, the IF-Cbl complex is degraded into the peroxisome with the recycling of cubam protein on the apical side, degrading IF and releasing Cbl from the baseline side due to the MRP1 transport protein. Here Cbl is readily associated with blood carriers [58]. In the blood stream, the cobalamins are transported by the trans-cobalamin II carrier (TCII) in a complex defined as holotranscobalamin II (HTCII) and considered the active Cbl form circulating in blood, as this transporter is highly specific to the molecules with vitamin activity [59]. Presumably, the TCII is secreted by endothelial cells of the cardiovascular system [60]. HTCII represents only a small percentage of the circulating Cbl (5%–20%). The remaining Cbl is linked to another more aspecific carrier named haptocorrin or transcobalamin III (HC), an isoform of the R-binder molecule [61]. While all cells have a specific receptor for HTCII called TCb1R, HC receptors have been discovered only in hepatocytes. It is thought that the function of this transporter is inactive analog removal through reverse transport to the liver and intestinal release from bile salts [62]. The HTCII complex that is not removed from the blood stream by the cell membrane protein TCb1R is filtered by the kidney, and reabsorbed in the proximal tubule through megalin protein-mediated reuptake [63]. The binding specificity of the different carriers for vitamin, high for IF and TCII and low for R-binder and HC, is necessary to reduce the absorption of a non-vitamin or degraded Cbl molecules that may saturate the transport system and affect vitamin absorption. Thus, the function of some carriers acquires the role of scavenger of the non-active corrinoid analogs. The TCb1R-HTCII complex is internalized in peripheral cells and degraded in the peroxisome with receptor recycling and proteolysis of TCII, including the release of Cbl in the cytosol due to transporters LMBD1 and ABCD4 [51,64,65]. Here, chaperon CblC removes upper ligands (methyl, hyroxyl, cyanide, or adenosyl), preparing Cbl for methylation and adenosylation [37,66,67].

Active absorption is severely limited. It is estimated that the absorptive capacity of Cbl is 1.5–2 μg per meal and depends on the maximum saturation of the cubam pool [68]. Conditions that reduce the secretion or binding efficiency can inhibit this amount significantly, as occurs with aging, with the chronic use of proton pump inhibitors (PPI) in Gastro-Esophageal Reflux Disease (GERD) or with gastric dysfunctions such as atrophic gastritis [69]. There is, however, a passive diffusion across the mucosal epithelium. This system is concentration-dependent and it is estimated that about 1% of Cbl follows this route. The aspecific transport is gradient-dependent and may be significant in the use of high oral doses (0.5–1 mg), with a good efficacy also in persons with limited capacity for active absorption, such as in cases of pernicious anemia or gastric atrophy [70,71]. Enterohepatic circulation is essential for the efficient absorption of Cbl. In fact, an excess of Cbl not internalized by cells is also secreted with bile and reabsorbed by the IF transport pathway [72]. Infants born to mothers with an adequate intake of Cbl show reserves of about 25 μg at birth [73]. Due to the intense activity of cell replication in tissue expansion, breast milk should continue to provide Cbl linked to a corrin, from which it becomes separated through the proteolytic action of the child’s gastrointestinal enzymes [74].

## 5. Assessment and Diagnostic Markers

There is no reference method or gold standard regarding Cbl deficiency and the clinical signs could be hematological, neurological and/or biochemical [75]. The deficiency of Cbl is defined by the serum concentrations <110 pmol/L [76,77], <127 pmol/L [78], <148 pmol/L [79,80], <150 pmol/L [81,82,83], <156 pmol/L [84,85], <220 pmol/L [86], <250 pmol/L [8]. The Institute of Medicine defined 120–180 pmol/L as a depletion range [25]. With such heterogeneous deficiency criteria, there is a risk of ignoring conditions of medical relevance. Moreover, even at concentrations above the cutoffs of 156 pmol/L (a commonly used cutoff), deficiency conditions may already be present [18,87]. The predictive strength of blood concentration of total Cbl is very low and insufficient for a diagnosis of deficiency, which may result in loss of memory, personality disturbance, emotional liability and psychosis even with a low-normal Cbl blood concentration [88]. In fact, total Cbl does not take into account the ratio between HTCII and Cbl bound to haptocorrin. The detection of HTCII can provide useful information on the immediate availability of dietary Cbl [63]. The blood concentration of HTCII, like those of total Cbl, respond quickly to increased intake and thus can be misleading regardless of cellular restoration while using supplements [75]. HTCII is a more effective diagnostic marker compared to total Cbl but may be affected by the use of contraceptives as well as kidney or liver disease [89,90]. The sufficiency cutoff for HTCII is more homogeneously defined in literature as a plasma concentration above 35 pmol/L [18,82,84,91]. A Cbl deficiency at the cellular level is manifested through an accumulation of the intermediate products of the metabolic pathways, in which it participates as a coenzyme. The increased plasma concentration of HCY and urinary or serum MMA can provide more detailed information on the deficiency condition. As for serum Cbl, the criteria for an excess of HCY are heterogeneous: >15 μmol/L [78,80,81,83,85,92], >12 μmol/L [18,76,77,84,86,91,93,94], >10 μmol/L [8]. In addition, there are different criteria of deficiency for serum MMA cutoffs ranging from >271 nmol/L to >376 nmol/L, with the former being most prevalent [18,81,82,84,85,93,94,95]. Both blood markers, MMA and HCY, may be altered by renal failure and it is therefore useful to test glomerular function with creatinine [96]. The use of urinary MMA standardized for creatinine, although used less, may reduce the risk of an incorrect diagnosis [97]. Normally, the cutoff used for the assessment of Cbl deficiency by urinary MMA is >4 μg per mg of creatinine [98,99]. The increase of MMA can also be caused by intestinal bacterial overgrowth through the conversion of propionic acid produced by the human intestinal microbiota [100]. Given the high intake of vegetables in a vegetarian diet, it is unlikely that a rise of HCY is dependent on folate deficiency. It is likely that in fortification areas, where flour is enriched with folic acid, the main reason for the rise in plasma HCY is Cbl depletion [79]. The EFSA defined Cbl deficiency with serum Cbl below 140 pmol/L, serum MMA above 750 nmol/L, plasma HCY above 15 μmol/L and serum HTCII below 21–45 pmol/L [24]. In the Third National Health and Nutrition Examination Survey (NHANES III), the cutoffs for the diagnosis of hyperhomocysteinemia were defined as HCY above 11.4 μmol/L and 10.4 μmol for men and women, respectively [101]. The different values of diagnostic markers discussed are summarized in Table 1, Table 2 and Table 3.

Some hematological markers can help to identify a shortage of vitamins, discriminating against red cell disorders as iron deficiency anemia, heterozygous thalassemia or other chronic diseases. The MCV and red cell distribution width (RDW) may increase in case of reduced availability of Cbl because of underlying hematopoietic alterations [102,103].

Herbert defined various stages of deficiency with the use of multiple markers as summarized in Table 4 [72]:

Unfortunately, some of the markers listed are not part of routine laboratory investigations and it is often difficult to locate diagnostic facilities that can perform all these tests.

Deficiency states could be boosted by impaired absorption. The Schilling test is a method, now rarely used, that was developed in 1953 (thanks to the availability of labeled Cbl) and applied in the diagnosis of food Cbl malabsorption [104]. This method quantifies the fraction of orally ingested labeled Cbl excreted in urine. Even if the Schilling test was considered the gold standard in the diagnosis of a Cbl deficiency, the use of radioactive material and misleading results in patients with antibodies against IF make it less suitable for clinical practice [105].

## 6. Status among Vegetarians

In Western countries, the intake of Cbl in the general population appears to be above the estimated requirements [19,21,24]. Its need during pregnancy and lactation increase due to the expansion of tissues and delivery to the fetus or the newborn. Thus the recommended dietary allowance has been determined at 2.6 μg/day and 2.8 μg/day for pregnancy and lactation, respectively [19,20]. The EFSA, however, settled on a safer amount of AI at 4.5 μg/day and 5 μg/day for pregnant and breastfeeding women, respectively [24]. A Cbl deficiency may occur by absorption alteration or nutritional insufficiency. Deficiencies are common in the elderly as a result of secondary hypochlorhydria due to drug treatment or a physiological alteration of the gastrointestinal mucosa itself [106]. The malabsorption can take place in cases of gastric or ileal resections, inflammatory bowel disease or for genetic defects in transport and cellular trafficking proteins [47,51,107]. The shortage of Cbl for low or no intake from food has been documented in low-income populations with poor nutritional status or in the case of vegetarians, with the first reports among vegetarian Indian people and Seventh-Day Adventist Church members [108,109]. Recent studies reported low serum cobalamin among vegetarians [103]. A deficiency in 11%–90% of elderly, 62% of pregnant women, 25%–86% of children, and 21%–41% of adolescents has been documented [110]. In a systematic review of literature based on the blood concentration of Cbl among vegetarians, a deficiency was present ranging from 0% to 86.5% among adults and elderly, up to 45% in infants, from 0% to 33.3% in children and adolescents, and from 17% to 39% among pregnant women [111]. The use of supplements or fortified foods seems to prevent deficiencies, indicating that a well-planned plant-based diet has proven to be adequate and sustainable [112,113]. However, despite the use of fortified foods, deficiency over a period of five years could occur, demonstrating a continuing insufficient intake or a possible decline in the absorptive capacity due to aging [114]. In all likelihood, even when supplementation occurs, it is possible that concentrations sufficient to avoid the reduction of body stock in the liver, blood and kidney cannot be reached. The liver is the main reservoir with a capacity of around 1–1.5 mg of Cbl [114].

Supplementation is often avoided due to preconceptions and aversion to products which are thought to be artificial, or due to the myth that the shortage will manifest itself only in rare cases after many years of ceased intake, an idea also supported by some researchers [115]. Although the shortage is documented in the macrobiotic community, many feel reluctant to use supplements, fortified foods, and more generally, processed foodstuffs [116].

The concomitant use of more specific markers enables a more detailed diagnosis. In adult German vegetarians, Cbl deficiency was present in 58%–66% or 61%–72% of participants if both criteria HTCII/MMA or HTCII alone were adopted, respectively [84,91,93,95]. Cbl deficiency leads to the accumulation of HCY, a molecule linked independently to the risk of cardiovascular disease (CVD), endothelial dysfunction and diabetes [117,118]. For each increment of 5 μmol/L of plasma HCY, there was a 20% increase in the risk of coronary heart disease (CHD) [119]. Concentrations of HCY among vegetarians seem to be higher and this correlates negatively with cardiovascular risk [54,76,103]. In a qualitative and quantitative review of the literature, it emerged that hyperhomocysteinemia with or without Cbl hipovitaminosis is a compelling risk factor for dementia [120]. In a meta-analysis on the status of vegetarians compared with omnivores by plasma HCY, six cohort case studies and 11 cross-sectional studies with a total of 3230 participants were analyzed, and an inverse relationship between HCY and Cbl for all diets was detected. Gradual worsening of both markers was evident from omnivores to VN with intermediate values in LV/LOV [54]. The HCY mechanisms of action are still unclear, but several theories have been proposed, including the initiation of the atherogenic process of reactive oxygen species (ROS) formation, inhibition of nitric oxide (NO) synthesis and influences on aortic calcification [121,122,123]. A recent meta-analysis of genome-wide association studies examined 18 single-nucleotide polymorphisms associated with total HCY concentrations [124]. A Mendelian randomization strategy was used to find no association between total HCY and the risk of coronary artery disease in genetic variants. These data may mitigate a causal correlation between HCY and CVD, but total blood HCY could be a useful marker for Cbl deficiency. When HCY, HTCII or MMA were used alone as diagnostic markers, the prevalence of deficiencies was greater in VN than in LOV/LV [18,76,77,85,91]. Although in the past it was thought that only the VN were at risk of vitamin deficiencies, recent studies indicate that even the LOV are at risk [125,126,127]. Herrmann et al. found that deficiency rates among LOV/LV and VN were 32% and 43%, respectively [85]. Supplementation in LOV/LV was effective in reducing deficiency rates from 68% to 31%, but the amounts were still insufficient [18]. Also the increase of MCV and RDW, associated with the lack of Cbl, leads to the increased cardiovascular risk [128,129]. Neurological manifestations of vitamin deficiency can also occur in the absence of anemia [130]. If the repletion comes late, the myelin degeneration caused by deficiency can also be irreversible [131]. In a review of 89 case studies of Cbl deficiency, 13 of which were vegetarianism related, neurological manifestations included progressive spastic paraparesis, acute onset of irrelevant speech and inability to comprehend, involuntary movements of the upper extremities, unsteady gait, acute onset of dizziness, ataxia, vomiting, mildly impaired cognitive functions, disorientation in time and short-term memory loss [132]. Psychiatric abnormalities and dermatological and oral manifestations were also found [132]. In the absence of Cbl sources, the decline of Cbl in blood is already evident during the first five years after the adoption of a vegetarian diet even without taking into account more specific markers, dispelling the myth that the shortage appears only after a very long time [101]. The first signs of a deficiency can occur as early as two years after Cbl intake has ceased [95,97]. The risks related to shortage are also frequent in children born to vegetarian mothers with inadequate reserves [133]. A depleted status among vegetarian women may be responsible for Cbl deficiency in infants, as well as a failure to thrive, low acceptability of solid foods and delays in neurodevelopment [74]. Moreover, there are indications that an inadequate Cbl status during pregnancy will affect fetal programming mechanisms, resulting in an increase of adiposity and insulin resistance in the offspring at six years, as well as for the mother herself [134,135]. The role of one-carbon metabolism of non-communicable diseases in fetal growth has been discussed [136]. The influence of Cbl deficiency during pregnancy and breastfeeding is so considerable that even a dietary restriction in the short term can result in an inadequate state in the infant [137]. In a pooled analysis of 48 cases of infant deficiency, there was a remarkable similarity in clinical symptoms between maternal veganism and maternal pernicious anemia [74].

## 7. Supplementation and Fortification

Supplements have been demonstrated as efficient in the restoration of Cbl blood concentration [97,138]. Currently, the official position of associations and government agencies is categorical and unequivocal: in the case of a vegetarian diet, including LOV, LV and OV, supplementation of Cbl is required [11,13]. Cbl concentration per 100 g of cow’s milk, dairy products and chicken eggs ranged from 0.5 to 0.4 μg, from 4.2 to 3.6 μg, and from 2.5 to 1.1 μg, respectively [139,140]. Taking into account the losses during cooking and the specific absorption rate, these quantities are not sufficient to ensure the daily intake in a balanced diet [141]. The microorganisms generally used for large-scale productions of cobalamin are *Propionibacterium freudenreichii*, *P. shermanii* and *Pseudomonas dentrificans* [142]. The World Health Organization (WHO) and the Food and Agriculture Organization (FAO) Expert Consultation have jointly released a guide for nutrient calculation procedures for the necessary quantities when fortifying foods [143]. In some countries, certain foods are fortified, such as breakfast cereals, with Cbl. However, the quantities used are quite variable and the consumption of such foods cannot guarantee sufficiency in the absence of other sources [25,139]. Some researchers show that the daily intake levels used are insufficient to ensure proper Cbl intake in population subgroups at risk, and they recommended a mandatory program of fortification of flour with folic acid, as is currently the norm in Canada and the USA [144,145]. Although folic acid in the blood seems high among vegetarians, it can bring about a subcellular deficiency as a result of the “folate trap” mechanism, in which the absence of Cbl blocks folate in the form of 5-methyltetrahydrofolate. This occurrence results in the blockage of the methyl group transfer to the substrate. The folate trap can mask a possible silent functional deficiency of folic acid, even with high folate serum concentration [146]. The vegetarian diet, rich in folacin, may mask hematological symptoms, so Cbl deficiency may only be evident due to neurological signs in the late stages, such as neuropsychiatric abnormalities, neuropathy, dementia and, albeit rarely, atrophy of optic nerves [85,130]. Usually hematologic manifestations and anemia precede neurologic signs, which are more severe and mostly irreversible [147,148]. The response to treatment is inversely proportional to the severity of the deficiency state and to the latency of intervention [130]. Cbl used in fortified foodstuffs and in supplements is in crystalline form. There are different products containing Me-Cbl, Ado-Cbl and H-Cbl, either as a supplement or as pharmaceutical compositions.

Cn-Cbl is the most used form due to its high stability, cost effectiveness and safety of use [45]. At present, a tolerable upper intake level (TUIL) for Cbl from food or supplements was not defined, as the published data are insufficient in determining toxicity events. An accumulation and an excess of absorption are highly unlikely, in fact Cbl is a water-soluble molecule that requires a specific transport system which is easily saturated [19,149]. Its safety has been demonstrated through the use of an ultra-high parenteral dose of 25 mg daily for 10 days followed by 25 mg monthly for five months [150]. Cn-Cbl is the most common form used in the literature and in supplement formulation. Furthermore, it is the only compatible form in fortification thanks to its decent stability when heated [30]. In rare cases of genetic defects in peroxisome activation enzymes, the use of provitamin forms may not be recommendable [45]. At high doses such as 1–2 mg, about 10 μg is absorbed through non-specific internalization, functioning also in malabsorption diseases [151]. The therapeutic administration of oral Cbl has proven to be as effective as intramuscular administration [152]. This is very useful, as intramuscular administration is far more expensive and rather painful for the patient, as well as not being free from complications [153]. Recently, it was debated whether the coenzymes Me-Cbl and Ado-Cbl can be more efficient; that is, as a ready-to-use form that does not require a prior conversion [154,155]. The use of these more expensive formulations is not necessarily justified. In the literature, evidence of efficacy and safety is scarce. Moreover, a more responsive and less stable H-Cbl isoform may not reach the target [45]. If all forms of Cbl follow the same path of reduction-oxidation in the cell, the potential superiority of the coenzyme forms will not exist [45]. Since the crystalline form of Cbl is not bound to food proteins, the bioavailability in supplements is equal, if not superior. The Institute of Medicine (IOM) considers that naturally occurring Cbl in food is absorbed by 50% in healthy adults [25].

The vegetarian diet is very high in fiber, which may reduce the ability to absorb some nutrients efficiently [156]. An excess of fiber in the diet could disrupt the re-absorption of the Cbl mechanism through enterohepatic circulation, although there is no evidence to confirm that this happens in humans [157]. While there is not a higher incidence of anemia among vegetarians than in omnivores, in some cases iron intake may be inadequate [158]. Due to the low bioavailability of inorganic iron, the Institute of Medicine decided that the iron requirement is 1.8 times higher in a vegetarian diet [19]. Lower concentrations of iron can act on the gastric mucosa which favors atrophic gastritis, reducing the absorption capacity of Cbl via the intrinsic factor [110,159]. Although bone fractures among vegetarians are not statistically more frequent than in omnivores, a plant-based diet can result in lower calcium intake, especially among the VN [160]. The internalization of the IF-Cbl complex takes place thanks to a mechanism that requires calcium to function properly. A lower intake of calcium in the diet can inhibit the Cbl endocytosis mechanism in the distal ileum [161]. It was proposed that the metformin pharmacological treatment can alter the Ca-dependent uptake absorption of Cbl [162]. There are some indications that there is a correlation between polyunsaturated fatty acids of the series 3 (*n*3PUFA), especially long-chain (LC), and blood concentrations of HCY [163,164]. From preliminary data, the increased intake of LC *n*3PUFAs reduced plasma HCY with statistical significance [165,166,167]. Frequently, the vegetarian diet provides a high intake of *n*6PUFA and reduced contributions from *n*3PUFA, especially LC *n*3PUFA [168]. In addition to the possible link with HCY, excess *n*6PUFA can directly increase cardiovascular risk through the generation of proinflammatory eicosanoids and platelet aggregation [169]. For the above reasons, vegetarians should not underestimate Cbl intake through the appropriate use of supplements, while maintaining a balanced diet according to individual needs.

Absorption of Cbl from supplements depends on the dose and frequency of the intake [170]. The absorption capacity depends on the saturable active transport and on the efficiency of the aspecific route. The consumption of oral doses of 1 μg, 10 μg, 50 μg, 500 μg, 1000 μg, are absorbed with an efficiency of 56%, 16%, 3%, 2%, 1.3%, respectively [151]. The presence of Cbl inside the human gut is not a sufficient amount in terms of a daily intake, since it cannot be absorbed through the specific transport that requires binding to the transporters. In addition, 98% of corrinoid compounds synthesized by the microbiota found in human feces appear to be inactive [171].

Using multivitamins can be inefficient and counterproductive for the supplementation of Cbl. The Cbl can be degraded in the presence of vitamin C and copper with the formation of inactive by-products. These compounds can inhibit the transport system interacting with transporter proteins [172,173]. Nutritional yeast fortified with Cbl is available in the USA, though its use may be less effective than supplements, in the case of deficiency [97].

## 8. Vegetable Sources of Vitamin B12 and Future Research

The quantification of the Cbl content in foodstuff can be performed by various analytical systems, some of which are not able to distinguish between active and pseudo-Cbl analog forms. The recent use of more accurate systems, such as gas chromatographic methods or IF-based chemiluminescence, clarified that often the content of Cbl in food may have been overestimated [26].

Vegetables like broccoli, asparagus and bean sprouts contain only traces of Cbl [174]. The presence of 0.1–1.2 μg/100 g in tea leaves is not enough to consider tea as an adequate source for daily intake [175]. The most commonly eaten mushrooms in Europe, such as porcini and *pleurotus*, do not contain relevant amounts of Cbl [31]. However, an Italian study has shown that selected types of oyster mushrooms grown in the southern areas of Italy show a wide range of concentrations of Cbl from 0.44 to 1.93 μg/100 g, as detected by ELISA immunoassay. The highest concentration was found in the species *Pleurotus nebrodalis*, typical of the mountain areas in central Sicily [176]. Less common mushrooms such as *Craterellus cornucopioides* or *Cantharellus cibarius* may contain 1.09–2.65 μg/100 g [177]. Shiitake mushrooms, popular among vegetarians, can contain up to 5.61 ± 3.9 μg of Cbl per 100 g of dry weight (mostly in active form), although with great variability [178]. Even in this case, although a portion of 50 g of dried shiitake could be adequate to achieve the daily requirement, it is unlikely that this will happen daily. Among the most widely used edible seaweeds, *Enteromorpha* sp. and *Porphyra* sp. (also known as nori) contain relevant amounts of Cbl ranging from 32.3 to 63.6 μg/100 g [179]. In vitro tests are promising, but there are not enough human clinical trials to consider the use of seaweed as favorable in vitamin provision [180,181]. In a clinical trial of six vegan children, the daily use of nori seaweed seemed to prevent Cbl deficiency, measured via serum Cbl [182]. In disagreement with these data, Dagnelie et al. found no positive effects in using nori seaweed and spirulina on Cbl-deficient children [183]. The Cbl content of other edible macroalgae is negligible and approximately zero [184]. In a pilot study, supplementation with Klamath microalgae (*Aphanizomenon flos-Aque*) improved Cbl status among 15 vegan subjects, assessed by serum Cbl and plasma HCY in a three-month open-label intervention [185]. Klamath contains about 32 μg/100 g of Cbl but Watanabe et al. extracted only a pseudovitamin analog from Klamath [186]. *Chlorella pyrenoidosa* is a microalgae frequently used as a supplement [187]. Corrinoid content in micro and macroalgae depends on an exogenous uptake due to the association with microorganisms responsible for the biosynthesis of Cbl [33]. In a pilot study of 17 LOV/VN, 9 g of *Chlorella* for 30–60 days were effective in mitigating Cbl deficiency, although these quantities are not compatible with a daily intake over the time period and the study was not independent [188]. Cbl in commercial preparations can be highly variable and still lack sufficient clinical trials on humans to verify the viability of use [189]. Several edible cyanobacteria, such as *Spirulina platensis* and *Nostoc* sp., contain significant amounts of corrinoids, many of which appear to be pseudovitamin [190]. Characterization with sensitive methods able to discriminate from different corrinoid compounds showed that the concentration of Cbl in spirulina was 127–244 μg per 100 g of dry weight, about 80% of which were non-vitamin compounds [191]. At present, cyanobacteria cannot be considered a reliable source of Cbl [190,192,193]. Some fermented vegetable foods, such as sauerkraut, natto and tempeh, can have significant amounts of Cbl. It is unlikely that their daily use in Western countries represents a stable source of Cbl. The presence of Cbl in these foods depends on environmental bacteria randomly present in the fermentative microorganism pool [194]. It is very difficult to standardize the content from one product to another as they are subject to wide variation. Tempeh, for example, during the fermentation of soy beans can develop Cbl in a range between 0.7 to 8 μg per 100 g [195]. Other fermented soy foodstuff has only trace amounts of Cbl [196,197]. In sauerkraut production, the addition of *Proprionibacteria* sp. to cabbage may boost Cbl concentration up to 7.2 μg/100 g [198]. The use of organic fertilizer can increase the content of Cbl in spinach leaves up to 0.14 μg/100 g. However, the quantity of spinach that needs to be ingested in order to satisfy the daily requirement would be prohibitive [199]. Treatment with cyanocobalamin during the sprouting of daikon or through hydroponic techniques in lettuce may increase retention of Cbl and delivery through plant foods [200,201]. Promising data has indicated production of soy yogurt with an enhanced production of Cbl up to 18 μg/L, as detected with the HPLC method [202]. Cbl content in vegetable foods are summarized in Table 5.

## 9. Conclusions

The choice of limiting or removing foods of animal origin from the diet is increasing in popularity due to ethical, environmental and health reasons, posing doubts over whether a number of these restrictions could be detrimental or useful [203]. The vegetarian diet can be sustainable at all stages of life and in all physiological conditions, including infancy, pregnancy, lactation, senescence and sports [13]. Compared to non-vegetarians, vegetarians have reduced body mass index (BMI), serum cholesterol, serum glucose and blood pressure with a lower mortality rate due to ischemic heart disease (IHD) [17,204]. However, underestimating the correct supplementation of cobalamin (Cbl) can nullify these benefits [103]. It is also necessary that the diet be balanced and nutritionally adequate to reduce the risks of other deficiencies which could indirectly affect the absorption of Cbl. Studies on the use of plant foods to increase the Cbl intake are promising, but still require more data. Some seaweed, mushrooms and fermented foods can be a useful source of Cbl, but the data are still insufficient and production is too heterogeneous. The standardization of Cbl-rich plant foods may be useful in preventing vitamin deficiency, overcoming the frequent ideological barriers on supplementation. The use of fortified toothpaste could be a promising alternative to the fortification of flour, resolving the possible reduction of vitamins during cooking [205]. Studies of efficacy in maintaining vitamin sufficiency with different Cbl forms are absent. More trials with vegetarian people using supplements or fortified foods are needed to better explain the efficacy of different strategies of cobalamin uptake, focusing on the best dosages, media or foods, if reliable. Future research on the half-life of Cbl in various human body districts after the intake of different vitamin B12 supplements (H-Cbl, Ado-Cbl, Me-Cbl, Cn-Cbl) would be very useful in helping us understand Cbl utilization and needs, especially in vegetarians. At present, there is no international consensus for supplementation in vegetarians. According to Carmel, a single oral dose of 50 μg, 500 μg or 1000 μg will be absorbed at an amount of 1.5 μg, 9.7 μg or 13 μg, respectively [151]. To meet the daily requirement of Cbl, one oral dose of 50–100 μg daily or 2000 μg weekly divided into two oral cyanocobalamin doses could be sufficient to meet the needs of 2.4 μg/day for healthy vegetarian adults, taking into account the efficiency of absorption and the passive route. In 1988, Herbert stated that vegetarians’ Cbl requirements could be satisfied by 1 μg tablet of vitamin B12 per day, based on human reabsorption through enterohepatic circulation [32]. This quantity is fairly low in respect to the proposed values for Cbl Dietary Reference Intake (DRI) in the general population [19,20]. Cyanocobalamin is the most economical—and historically the most used form—rendering it suitable for safe daily use [45]. There were no apparent substantial differences between the absorption of sublingual and oral forms [152,206]. However, oral dissolution could be critical in the secretion of the salivary R-binder and its subsequent bond. Since the Cbl would not be dissolved, about 88% could be not absorbed [54]. Since the development of a Cbl deficiency can also be observed among the LOV, the use of a supplement is necessary, regardless of the type of vegetarian diet [110]. In cases of malabsorption, such as hypochlorhydria or functional deficit, such amounts might be insufficient [151,207]. With senescence, the lowering of gastric secretion reduces the direct absorption capacities due to reduced release of IF and the impairment of active transport, but not of the passive transport. At the same time, the natural gastric barrier decreases with consequent risk of gastrointestinal bacterial overgrowth and competing for the use of ingested Cbl [69]. If rare genetic defects of cellular trafficking and processing proteins exist, the choice of alternative forms of Cbl, such as Me-Cbl or H-Cbl could improve the effectiveness of supplementation [154,208,209,210]. It was speculated that the use of Cn-Cbl is unsuitable for supplementation among smokers, because it represents a form preferentially excreted for the purpose of hydrocyanic acid removal [211,212]. In these cases, the use of H-Cbl may be desirable, although it is yet to be confirmed [213,214]. The data currently available do not permit an estimate of a maximum intake level for cobalamin. High levels of administration in particular lead to rare adverse events, mostly dermatological, such as pruritus, rash and skin eruptions [215]. The sufficiency of bodily stocks should not be a reason to delay supplementation, in light of the fact that the manifestation of the deficiency can occur through often irreversible neurological signs. The current data do not support the theory that vitamin deficiency needs 20–30 years to be manifested [125]. Nevertheless, future studies should take into account the possibility of supplementation even in a subclinical condition, as was already done for other deficiencies [216,217], which may become overt during the time-period [218,219]. The use of multiple diagnostic markers can facilitate the correct assessment of the status and allow a more sound decision in relation to the administration plan. The habit of using only one or a few of the multiple markers available implies that the deficiency status of Cbl among vegetarians, as among omnivores, may have sometimes been underestimated. Moreover, cobalamin displays other functions, not strictly metabolic, that could be lacking when deficient. A vitamin B12 deficiency could be related to oxidative stress markers like plasma glutathione, malondialdehyde and serum total antioxidant capacity, which could contribute to a neurophysiological disturbance [220]. Furthermore, Cbl, particularly H-Cbl, can act as a detoxifying agent, removing potentially dangerous cyanide molecules from the body [212].

## Figures and Tables

**Table 1:** Vitamin B12 deficiency criteria.

Value	Country	References
<110 pmol/L	Austria	[76,77]
<127 pmol/L	Italy	[78]
<148 pmol/L	USA	[79]
<148 pmol/L	India	[80]
<150 pmol/L	India	[81]
<150 pmol/L	USA	[82]
<150 pmol/L	Turkey	[83]
<156 pmol/L	Germany/The Netherlands	[84]
<156 pmol/L	Germany	[85]
<220 pmol/L	Slovakia	[86]
<250 pmol/L	Germany	[8]

**Table 2:** Hyperhomocysteinemia criteria.

Value	Country	References
>15 μmol/L	Italy	[78]
>15 μmol/L	India	[80,81]
>15 μmol/L	Turkey	[83]
>15 μmol/L	Germany	[85]
>15 μmol/L	Taiwan	[92]
>12 μmol/L	Germany/The Netherlands	[18,84,94]
>12 μmol/L	Austria	[76,77]
>12 μmol/L	Slovakia	[86]
>12 μmol/L	Germany	[91,93]
>10 μmol/L	Germany	[8]

**Table 3:** Methylmalonic acid values for vitamin B12 deficiency.

Value	Country	References
>260 nmol/L	India	[81]
>271 nmol/L	Germany/The Netherlands	[18,84,94]
>271 nmol/L	Germany	[85,93]
>271 nmol/L	Germany/Oman	[95]
>376 nmol/L	USA	[82]

**Table 4:** Herbert’s stratification for vitamin B12 deficiency [72].

Stage	Markers	Interpretation
I	HTCII	Blood and cellular reserves reduced with low HTCII
II	HC	Low concentrations of HC
III	HCY/MMA	Functional unbalanced with high concentrations of HCY and MMA
IV	MCV/Hb ^1^	Clinical signs like high MCV, low Hb and macroovalocytosis

^1^ Hb: Hemoglobin.

**Table 5:** Cobalamin content in vegetable foods.

Foods	μg/100 g	Assay	References
Tea leaves	0.1–1.2	Microbiological	[175]
Tea leaves	0.046–0.859	IF-Chemiluminescence	[175]
Mushrooms (*Porcini*, *Pleurotus*)	0.01–0.09	LC/ESI-MS/MS	[177]
Mushrooms (*C. cornucopioides*, *C. cibarius*)	1.09–2.65	LC/ESI-MS/MS	[177]
Mushrooms (*Pleurotus* spp. from Sicily)	0.44–1.93	ELISA	[176]
Mushrooms (Shiitake)	3.95–5.61	LC/ESI-MS/MS	[178]
Seaweed (Nori)	32.26–63.58	Microbiological	[179]
Seaweed (Nori)	25.07–69.20	IF-Chemiluminescence	[179]
Microalgae (Klamath)	31.06–34.27	IF-Chemiluminescence	[186]
Microalgae (*Chlorella*)	200.9–211.6	IF-Chemiluminescence	[189]
Cyanobacteria (Spirulina)	127.2–244.3	Microbiological	[191]
Cyanobacteria (Spirulina)	6.2–17.4	IF-Chemiluminescence	[191]
Cyanobacteria (*Nostoc*)	11	HPLC	[190]
Tempeh	0.7–8	Not specified	[195]
Tempeh	0.02–0.7	Microbiological	[196]
Sauerkraut	Up to 7.2	Microbiological	[198]

## References

[1] EURISPES 2016. http://eurispes.eu/content/rapporto-italia-2016-la-sindrome-del-palio.

[2] Larsson C.L., Johansson G.K. (2002). Dietary intake and nutritional status of young vegans and omnivores in Sweden. Am. J. Clin. Nutr..

[3] World Health Organization (2014). Global Status Report on Non Communicable Diseases 2014.

[4] Ferdowsian H.R., Barnard N.D. (2009). Effects of plant-based diets on plasma lipids. Am. J. Cardiol..

[5] Pettersen B.J., Anousheh R., Fan J., Jaceldo-Siegl K., Fraser G.E. (2012). Vegetarian diets and blood pressure among white subjects: Results from the Adventist Health Study-2 (AHS-2). Public Health Nutr..

[6] Barnard N.D., Katcher H.I., Jenkins D.J., Cohen J., Turner-McGrievy G. (2009). Vegetarian and vegan diets in type 2 diabetes management. Nutr. Rev..

[7] Tonstad S., Butler T., Yan R., Fraser G.E. (2009). Type of vegetarian diet, body weight, and prevalence of type 2 diabetes. Diabetes Care.

[8] Waldmann A., Koschizke J.W., Leitzmann C., Hahn A. (2005). German vegan study: Diet, life-style factors, and cardiovascular risk profile. Ann. Nutr. Metab..

[9] Key T.J., Appleby P.N., Davey G.K., Allen N.E., Spencer E.A., Travis R.C. (2003). Mortality in British vegetarians: Review and preliminary results from EPIC-Oxford. Am. J. Clin. Nutr..

[10] Key T.J., Fraser G.E., Thorogood M., Appleby P.N., Bera V., Reeves G., Burr M.L., Chang-Claude J., Frentzel-Beyme R., Kuzma J.W. (1999). Mortality in vegetarians and nonvegetarians: Detailed findings from a collaborative analysis of 5 prospective studies. Am. J. Clin. Nutr..

[11] U.S. Department of Agriculture, U.S. Department of Health and Human Services (2010). Dietary Guidelines for Americans 2010.

[12] American Dietetic Association (1988). Position of the American Dietetic Association: Vegetarian diets. J. Am. Diet. Assoc..

[13] Craig W.J., Mangels A.R., American Dietetic Association (2009). Position of the American Dietetic Association: Vegetarian diets. J. Am. Diet. Assoc..

[14] Allen L.H. (2008). Causes of vitamin B12 and folate deficiency. Food Nutr. Bull..

[15] O’Leary F., Samman S. (2010). Vitamin B12 in health and disease. Nutrients.

[16] Stollhoff K., Schulte F.J. (1987). Vitamin B12 and brain development. Eur. J. Pediatr..

[17] Li D. (2011). Chemistry behind Vegetarianism. J. Agric. Food Chem..

[18] Herrmann W., Schorr H., Obeid R., Geisel J. (2003). Vitamin B-12 status, particularly holotranscobalamin II and methylmalonic acid concentrations, and hyperhomocysteinemia in vegetarians. Am. J. Clin. Nutr..

[19] Otten J.J., Hellwig J.P., Meyers L.D. (2006). Dietary Reference Intakes: The Essential Guide to Nutrient Requirements.

[20] Società Italiana di Nutrizione Umana (2014). LARN: Livelli di Assunzione di Riferimento di Nutrienti ed Energia per la Popolazione Italiana.

[21] U.S. Department of Agriculture, Agricultural Research Service, Beltsville Human Nutrition Research Center, Food Surveys Research Group, U.S. Department of Health and Human Services, Centers for Disease Control and Prevention, National Center for Health Statistics What We Eat in America, NHANES 2009–2010. Nutrient Intakes from Food: Mean Amounts Consumed per Individual, by Gender and Age, in United States, 2009–2010. https://www.ars.usda.gov/SP2UserFiles/Place/80400530/pdf/0910/tables_1-40_2009-2010.pdf.

[22] Obeid R., Schorr H., Eckert R., Herrmann W. (2004). Vitamin B12 status in the elderly as judged by available biochemical markers. Clin. Chem..

[23] Yetley E.A., Pfeiffer C.M., Phinney K.W., Bailey R.L., Blackmore S., Bock J.L., Brody L.C., Carmel R., Curtin L.R., Durazo-Arvizu R.A. (2011). Biomarkers of vitamin B-12 status in NHANES: A roundtable summary. Am. J. Clin. Nutr..

[24] Panel on Dietetic Products, Nutrition, and Allergies (2015). Scientific opinion on dietary reference values for cobalamin (Vitamin B12). EFSA J..

[25] Institute of Medicine (1998). A Report of the Standing Committee on the Scientific Evaluation of Dietary Reference Intakes and Its Panel on Folate, Other B Vitamins, and Choline and Subcommittee on Upper Reference Levels of Nutrients, Food and Nutrition Board.

[26] Watanabe F. (2007). Vitamin B12 sources and bioavailability. Exp. Biol. Med..

[27] Bor M.V., Lydeking-Olsen E., Møller J., Nexø E. (2006). A daily intake of approximately 6 microg vitamin B-12 appears to saturate all the vitamin B-12-related variables in Danish postmenopausal women. Am. J. Clin. Nutr..

[28] Andrès E., Kurtz J.E., Perrin A.E., Maloisel F., Demangeat C., Goichot B., Schlienger J.L. (2001). Oral cobalamin therapy for the treatment of patients with food-cobalamin malabsorption. Am. J. Med..

[29] Peters R., Burch L., Warner J., Beckett N., Poulter R., Bulpitt C. (2008). Haemoglobin, anaemia, dementia and cognitive decline in the elderly, a systematic review. BMC Geriatr..

[30] Watanabe F., Yabuta Y., Tanioka Y., Bito T. (2013). Biologically active vitamin B12 compounds in foods for preventing deficiency among vegetarians and elderly subjects. J. Agric. Food Chem..

[31] Watanabe F., Yabuta Y., Bito T., Teng F. (2014). Vitamin B_12_-containing plant food sources for vegetarians. Nutrients.

[32] Herbert V. (1988). Vitamin B-12: Plant sources, requirements, and assay. Am. J. Clin. Nutr..

[33] Watanabe F., Abe K., Takenaka S., Tamura Y., Maruyama I., Nakano Y. (1997). Occurrence of cobalamin coenzymes in the photosynthetic green alga, *Chlorella vulgaris*. Biosci. Biotechnol. Biochem..

[34] Smith E.L. (1948). Purification of anti-pernicious anaemia factors from liver. Nature.

[35] Randaccio L., Geremia S., Demitri N., Wuerges J. (2010). Vitamin B12: Unique metalorganic compounds and the most complex vitamins. Molecules.

[36] Quadros E.V. (2010). Advances in the understanding of cobalamin assimilation and metabolism. Br. J. Haematol..

[37] Gherasim C., Lofgren M., Banerjee R. (2013). Navigating the B(12) road: Assimilation, delivery, and disorders of cobalamin. J. Biol. Chem..

[38] Cooper B.A., Rosenblatt D.S. (1987). Inherited defects of vitamin B12 metabolism. Annu. Rev. Nutr..

[39] Fenton W.A., Hack A.M., Willard H.F., Gertler A., Rosenberg L.E. (1982). Purification and properties of methylmalonyl coenzyme A mutase from human liver. Arch. Biochem. Biophys..

[40] Marsh E.N., Meléndez G.D. (2012). Adenosylcobalamin enzymes: Theory and experiment begin to converge. Biochim. Biophys. Acta.

[41] Scalabrino G., Carpo M., Bamonti F., Pizzinelli S., D’Avino C., Bresolin N., Meucci G., Martinelli V., Comi G.C., Peracchi M. (2004). High tumor necrosis factor-α in levels in cerebrospinal fluid of cobalamin-deficient patients. Ann. Neurol..

[42] Lipton S.A., Kim W.K., Choi Y.B., Kumar S., D’Emilia D.M., Rayudu P.V., Arnelle D.R., Stamler J.S. (1997). Neurotoxicity associated with dual actions of homocysteine at the *N*-methyl-d-aspartate receptor. Proc. Natl. Acad. Sci. USA.

[43] Hodgkin D.C., Kamper J., Mackay M., Pickworth J., Trueblood K.N., White J.G. (1956). Structure of vitamin B12. Nature.

[44] Chaouali N., Gana I., Dorra A., Khelifi F., Nouioui A., Masri W., Belwaer I., Ghorbel H., Hedhili A. (2013). Potential Toxic Levels of Cyanide in Almonds (*Prunus amygdalus*), Apricot Kernels (*Prunus armeniaca*), and Almond Syrup. ISRN Toxicol..

[45] Obeid R., Fedosov S.N., Nexo E. (2015). Cobalamin coenzyme forms are not likely to be superior to cyano- and hydroxyl-cobalamin in prevention or treatment of cobalamin deficiency. Mol. Nutr. Food Res..

[46] Banerjee R. (2006). B12 trafficking in mammals: A for coenzyme escort service. ACS Chem. Biol..

[47] Hannibal L., Kim J., Brasch N.E., Wang S., Rosenblatt D.S., Banerjee R., Jacobsen D.W. (2009). Processing of alkylcobalamins in mammalian cells: A role for the MMACHC (cblC) gene product. Mol. Genet. Metab..

[48] Gimsing P., Hippe E., Helleberg-Rasmussen I., Moesgaard M., Nielsen J.L., Bastrup-Madsen P., Berlin R., Hansen T. (1982). Cobalamin forms in plasma and tissue during treatment of vitamin B12 deficiency. Scand. J. Haematol..

[49] Herbert V., Sullivan L.W. (1964). Activity of Coenzyme B12 in Man. Ann. N. Y. Acad. Sci..

[50] Adams J.F., Ross S.K., Mervyn L., Boddy K., King P. (1971). Absorption of cyanocobalamin, coenzyme B 12, methylcobalamin, and hydroxocobalamin at different dose levels. Scand. J. Gastroenterol..

[51] Gailus S., Höhne W., Gasnier B., Nürnberg P., Fowler B., Rutsch F. (2010). Insights into lysosomal cobalamin trafficking: Lessons learned from cblF disease. J. Mol. Med..

[52] Wuerges J., Geremia S., Randaccio L. (2007). Structural study on ligand specificity of human vitamin B12 transporters. Biochem. J..

[53] Gruber K., Puffer B., Kräutler B. (2011). Vitamin B12-derivatives-enzyme cofactors and ligands of proteins and nucleic acids. Chem. Soc. Rev..

[54] Obersby D., Chappell D.C., Dunnett A., Tsiami A.A. (2013). Plasma total homocysteine status of vegetarians compared with omnivores: A systematic review and meta-analysis. Br. J. Nutr..

[55] Mathews F.S., Gordon M.M., Chen Z., Rajashankar K.R., Ealick S.E., Alpers D.H., Sukumar N. (2007). Crystal structure of human intrinsic factor: Cobalamin complex at 2.6-A resolution. Proc. Natl. Acad. Sci. USA.

[56] Muir M., Chanarin I. (1983). Separation of cobalamin analogues in human sera binding to intrinsic factor and to R-type vitamin B12 binders. Br. J. Haematol..

[57] Fyfe J.C., Madsen M., Højrup P., Christensen E.I., Tanner S.M., de la Chapelle A., He Q., Moestrup S.K. (2004). The functional cobalamin (vitamin B12)-intrinsic factor receptor is a novel complex of cubilin and amnionless. Blood.

[58] Beedholm-Ebsen R., van de Wetering K., Hardlei T., Nexø E., Borst P., Moestrup S.K. (2010). Identification of multidrug resistance protein 1 (MRP1/ABCC1) as a molecular gate for cellular export of cobalamin. Blood.

[59] Hippe E., Olesen H. (1971). Nature of vitamin B 12 binding. 3. Thermodynamics of binding to human intrinsic factor and transcobalamins. Biochim. Biophys. Acta.

[60] Quadros E.V., Rothenberg S.P., Jaffe E.A. (1989). Endothelial cells from human umbilical vein secrete functional transcobalamin II. Am. J. Physiol..

[61] Herzlich B., Herbert V. (1988). Depletion of serum holotranscobalamin II. An early sign of negative vitamin B12 balance. Lab. Investig..

[62] Ashwell G., Morell A. (1974). The dual role of sialic acid in the hepatic recognition and catabolism of serum glycoproteins. Biochem. Soc. Symp..

[63] Moestrup S.K., Birn H., Fischer P.B., Petersen C.M., Verroust P.J., Sim R.B., Christensen E.I., Nexø E. (1996). Megalin-mediated endocytosis of transcobalamin-vitamin-B12 complexes suggests a role of the receptor in vitamin-B12 homeostasis. Proc. Natl. Acad. Sci. USA.

[64] Nielsen M.J., Rasmussen M.R., Andersen C.B., Nexø E., Moestrup S.K. (2012). Vitamin B12 transport from food to the body’s cells—A sophisticated, multistep pathway. Nat. Rev. Gastroenterol. Hepatol..

[65] Rutsch F., Gailus S., Miousse I.R., Suormala T., Sagné C., Toliat M.R., Nürnberg G., Wittkampf T., Buers I., Sharifi A. (2009). Identification of a putative lysosomal cobalamin exporter altered in the cblF defect of vitamin B12 metabolism. Nat. Genet..

[66] Li Z., Gherasim C., Lesniak N.A., Banerjee R. (2014). Glutathione-dependent one-electron transfer reactions catalyzed by a B12 trafficking protein. J. Biol. Chem..

[67] Koutmos M., Gherasim C., Smith J.L., Banerjee R. (2011). Structural basis of multifunctionality in a vitamin B12-processing enzyme. J. Biol. Chem..

[68] Scott J.M. (1997). Bioavailability of vitamin B12. Eur. J. Clin. Nutr..

[69] Baik H.W., Russell R.M. (1999). Vitamin B12 deficiency in the elderly. Annu. Rev. Nutr..

[70] Berlin H., Berlin R., Brante G. (1968). Oral treatment of pernicious anemia with high doses of vitamin B12 without intrinsic factor. Acta Med. Scand..

[71] Elia M. (1998). Oral or parenteral therapy for B12 deficiency. Lancet.

[72] Herbert V. (1994). Staging vitamin B-12 (cobalamin) status in vegetarians. Am. J. Clin. Nutr..

[73] McPhee A.J., Davidson G.P., Leahy M., Beare T. (1988). Vitamin B12 deficiency in a breast fed infant. Arch. Dis. Child.

[74] Dror D.K., Allen L.H. (2008). Effect of vitamin B12 deficiency on neurodevelopment in infants: Current knowledge and possible mechanisms. Nutr. Rev..

[75] Schneede J., Ueland P.M. (2005). Novel and established markers of cobalamin deficiency: Complementary or exclusive diagnostic strategies. Semin. Vasc. Med..

[76] Elmadfa I., Singer I. (2009). Vitamin B-12 and homocysteine status among vegetarians: A global perspective. Am. J. Clin. Nutr..

[77] Majchrzak D., Singer I., Männer M., Rust P., Genser D., Wagner K.H., Elmadfa I. (2006). B-vitamin status and concentrations of homocysteine in Austrian omnivores, vegetarians and vegans. Ann. Nutr. Metab..

[78] Bissoli L., Di Francesco V., Ballarin A., Mandragona R., Trespidi R., Brocco G., Caruso B., Bosello O., Zamboni M. (2002). Effect of vegetarian diet on homocysteine levels. Ann. Nutr. Metab..

[79] Green R., Miller J.W. (2005). Vitamin B12 deficiency is the dominant nutritional cause of hyperhomocysteinemia in a folic acid-fortified population. Clin. Chem. Lab. Med..

[80] Naik S., Bhide V., Babhulkar A., Mahalle N., Parab S., Thakre R., Kulkarni M. (2013). Daily milk intake improves vitamin B-12 status in young vegetarian Indians: An intervention trial. Nutr. J..

[81] Refsum H., Yajnik C.S., Gadkari M., Schneede J., Vollset S.E., Orning L., Guttormsen A.B., Joglekar A., Sayyad M.G., Ulvik A. (2001). Hyperhomocysteinemia and elevated methylmalonic acid indicate a high prevalence of cobalamin deficiency in Asian Indians. Am. J. Clin. Nutr..

[82] Haddad E.H., Berk L.S., Kettering J.D., Hubbard R.W., Peters W.R. (1999). Dietary intake and biochemical, hematologic, and immune status of vegans compared with nonvegetarians. Am. J. Clin. Nutr..

[83] Karabudak E., Kiziltan G., Cigerim N. (2008). A comparison of some of the cardiovascular risk factors in vegetarian and omnivorous Turkish females. J. Hum. Nutr. Diet..

[84] Herrmann W., Obeid R., Schorr H., Geisel J. (2003). Functional vitamin B12 deficiency and determination of holotranscobalamin in populations at risk. Clin. Chem. Lab. Med..

[85] Herrmann W., Schorr H., Purschwitz K., Rassoul F., Richter V. (2001). Total homocysteine, vitamin B(12), and total antioxidant status in vegetarians. Clin. Chem..

[86] Krivosíková Z., Krajcovicová-Kudlácková M., Spustová V., Stefíková K., Valachovicová M., Blazícek P., Nĕmcová T. (2010). The association between high plasma homocysteine levels and lower bone mineral density in Slovak women: The impact of vegetarian diet. Eur. J. Nutr..

[87] Herrmann W., Geisel J. (2002). Vegetarian lifestyle and monitoring of vitamin B-12 status. Clin. Chim. Acta.

[88] Lindenbaum J., Healton E.B., Savage D.G., Brust J.C., Garrett T.J., Podell E.R., Marcell P.D., Stabler S.P., Allen R.H. (1988). Neuropsychiatric disorders caused by cobalamin deficiency in the absence of anemia or macrocytosis. N. Engl. J. Med..

[89] Riedel B., Bjørke Monsen A.L., Ueland P.M., Schneede J. (2005). Effects of oral contraceptives and hormone replacement therapy on markers of cobalamin status. Clin. Chem..

[90] Carmel R. (2002). Measuring and interpreting holo-transcobalamin (holo-transcobalamin II). Clin. Chem..

[91] Herrmann W., Obeid R., Schorr H., Geisel J. (2005). The usefulness of holotranscobalamin in predicting vitamin B12 status in different clinical settings. Curr. Drug Metab..

[92] Huang Y.C., Chang S.J., Chiu Y.T., Chang H.H., Cheng C.H. (2003). The status of plasma homocysteine and related B-vitamins in healthy young vegetarians and nonvegetarians. Eur. J. Nutr..

[93] Geisel J., Schorr H., Bodis M., Isber S., Hübner U., Knapp J.P., Obeid R., Herrmann W. (2005). The vegetarian lifestyle and DNA methylation. Clin. Chem. Lab. Med..

[94] Obeid R., Geisel J., Schorr H., Hübner U., Herrmann W. (2002). The impact of vegetarianism on some haematological parameters. Eur. J. Haematol..

[95] Herrmann W., Obeid R., Schorr H., Hübner U., Geisel J., Sand-Hill M., Ali N., Herrmann M. (2009). Enhanced bone metabolism in vegetarians—The role of vitamin B12 deficiency. Clin. Chem. Lab. Med..

[96] Stabler S.P., Lindenbaum J., Allen R.H. (1996). The use of homocysteine and other metabolites in the specific diagnosis of vitamin B-12 deficiency. J. Nutr..

[97] Donaldson M.S. (2000). Metabolic vitamin B12 status on a mostly raw vegan diet with follow-up using tablets, nutritional yeast, or probiotic supplements. Ann. Nutr. Metab..

[98] Kwok T., Cheng G., Lai W.K., Poon P., Woo J., Pang C.P. (2004). Use of fasting urinary methylmalonic acid to screen for metabolic vitamin B12 deficiency in older persons. Nutrition.

[99] Miller D.R., Specker B.L., Ho M.L., Norman E.J. (1991). Vitamin B-12 status in a macrobiotic community. Am. J. Clin. Nutr..

[100] Lindenbaum J., Savage D.G., Stabler S.P., Allen R.H. (1990). Diagnosis of cobalamin deficiency: II. Relative sensitivities of serum cobalamin, methylmalonic acid, and total homocysteine concentrations. Am. J. Hematol..

[101] Selhub J., Jacques P.F., Rosenberg I.H., Rogers G., Bowman B.A., Gunter E.W., Wright J.D., Johnson C.L. (1999). Serum total homocysteine concentrations in the third National Health and Nutrition Examination Survey (1991–1994): Population reference ranges and contribution of vitamin status to high serum concentrations. Ann. Intern. Med..

[102] Bessman J.D., Gilmer P.R., Gardner F.H. (1983). Improved classification of anemias by MCV and RDW. Am. J. Clin. Pathol..

[103] Pawlak R. (2015). Is vitamin B12 deficiency a risk factor for cardiovascular disease in vegetarians?. Am. J. Prev. Med..

[104] Schilling R.F. (1953). Intrinsic factor studies II. The effect of gastric juice on the urinary excretion of radioactivity after the oral administration of radioactive vitamin B12. J. Lab. Clin. Med..

[105] Chanarin I. (2000). Historical review: A history of pernicious anaemia. Br. J. Haematol..

[106] Herrmann W., Obeid R. (2008). Causes and early diagnosis of vitamin B12 deficiency. Dtsch. Arztebl. Int..

[107] Headstrom P.D., Rulyak S.J., Lee S.D. (2008). Prevalence of and risk factors for vitamin B(12) deficiency in patients with Crohn’s disease. Inflamm. Bowel Dis..

[108] Banerjee D.K., Chatterjea J.B. (1960). Serum vitamin B12 in vegetarians. Br. Med. J..

[109] Armstrong B.K., Davis R.E., Nicol D.J., van Merwyk A.J., Larwood C.J. (1974). Hematological, vitamin B 12, and folate studies on Seventh-day Adventist vegetarians. Am. J. Clin. Nutr..

[110] Pawlak R., Parrott S.J., Raj S., Cullum-Dugan D., Lucus D. (2013). How prevalent is vitamin B(12) deficiency among vegetarians?. Nutr. Rev..

[111] Pawlak R., Lester S.E., Babatunde T. (2014). The prevalence of cobalamin deficiency among vegetarians assessed by serum vitamin B12: A review of literature. Eur. J. Clin. Nutr..

[112] Schüpbach R., Wegmüller R., Berguerand C., Bui M., Herter-Aeberli I. (2015). Micronutrient status and intake in omnivores, vegetarians and vegans in Switzerland. Eur. J. Nutr..

[113] Mądry E., Lisowska A., Grebowiec P., Walkowiak J. (2012). The impact of vegan diet on B-12 status in healthy omnivores: Five-year prospective study. Acta Sci. Pol. Technol. Aliment..

[114] Rappazzo M.E., Salmi H.A., Hall C.A. (1970). The content of vitamin B12 in adult and foetal tissue: A comparative study. Br. J. Haematol..

[115] Rizzo N.S., Jaceldo-Siegl K., Sabate J., Fraser G.E. (2013). Nutrient profiles of vegetarian and nonvegetarian dietary patterns. J. Acad. Nutr. Diet..

[116] Van Dusseldorp M., Schneede J., Refsum H., Ueland P.M., Thomas C.M., de Boer E., van Staveren W.A. (1999). Risk of persistent cobalamin deficiency in adolescents fed a macrobiotic diet in early life. Am. J. Clin. Nutr..

[117] Van Guldener C., Stehouwer C.D. (2002). Diabetes mellitus and hyperhomocysteinemia. Semin. Vasc. Med..

[118] Homocysteine Studies Collaboration (2002). Homocysteine and risk of ischemic heart disease and stroke: A meta-analysis. JAMA.

[119] Humphrey L.L., Fu R., Rogers K., Freeman M., Helfand M. (2008). Homocysteine level and coronary heart disease incidence: A systematic review and meta-analysis. Mayo Clin. Proc..

[120] Werder S.F. (2010). Cobalamin deficiency, hyperhomocysteinemia, and dementia. Neuropsychiatr. Dis. Treat..

[121] Welch G.N., Loscalzo J. (1998). Homocysteine and atherothrombosis. N. Engl. J. Med..

[122] Shargorodsky M., Boaz M., Pasternak S., Hanah R., Matas Z., Fux A., Beigel Y., Mashavi M. (2009). Serum homocysteine, folate, vitamin B12 levels and arterial stiffness in diabetic patients: Which of them is really important in atherogenesis?. Diabetes Metab. Res. Rev..

[123] Mahalle N., Kulkarni M.V., Garg M.K., Naik S.S. (2013). Vitamin B12 deficiency and hyperhomocysteinemia as correlates of cardiovascular risk factors in Indian subjects with coronary artery disease. J. Cardiol..

[124] Van Meurs J.B., Pare G., Schwartz S.M., Hazra A., Tanaka T., Vermeulen S.H., Cotlarciuc I., Yuan X., Mälarstig A., Bandinelli S. (2013). Common genetic loci influencing plasma homocysteine concentrations and their effect on risk of coronary artery disease. Am. J. Clin. Nutr..

[125] Allen L.H. (2009). How common is vitamin B-12 deficiency?. Am. J. Clin. Nutr..

[126] Stabler S.P., Allen R.H. (2004). Vitamin B12 deficiency as a worldwide problem. Annu. Rev. Nutr..

[127] Antony A.C. (2003). Vegetarianism and vitamin B-12 (cobalamin) deficiency. Am. J. Clin. Nutr..

[128] Tonelli M., Sacks F., Arnold M., Moye L., Davis B., Pfeffer M., for the Cholesterol and Recurrent Events (CARE) Trial Investigators (2008). Relation between Red Blood Cell Distribution Width and Cardiovascular Event Rate in People with Coronary Disease. Circulation.

[129] Dabbah S., Hammerman H., Markiewicz W., Aronson D. (2010). Relation between red cell distribution width and clinical outcomes after acute myocardial infarction. Am. J. Cardiol..

[130] Ralapanawa D.M., Jayawickreme K.P., Ekanayake E.M., Jayalath W.A. (2015). B12 deficiency with neurological manifestations in the absence of anaemia. BMC Res. Notes.

[131] Brocadello F., Levedianos G., Piccione F., Manara R., Pesenti F.F. (2007). Irreversible subacute sclerotic combined degeneration of the spinal cord in a vegan subject. Nutrition.

[132] Rusher D.R., Pawlak R. (2013). A Review of 89 Published Case Studies of Vitamin B12 Deficiency. J. Hum. Nutr. Food Sci..

[133] Koebnick C., Hoffmann I., Dagnelie P.C., Heins U.A., Wickramasinghe S.N., Ratnayaka I.D., Gruendel S., Lindemans J., Leitzmann C. (2004). Long-term ovo-lacto vegetarian diet impairs vitamin B-12 status in pregnant women. J. Nutr..

[134] Yajnik C.S., Deshmukh U.S. (2008). Maternal nutrition, intrauterine programming and consequential risks in the offspring. Rev. Endocr. Metab. Disord..

[135] Knight B.A., Shields B.M., Brook A., Hill A., Bhat D.S., Hattersley A.T., Yajnik C.S. (2015). Lower Circulating B12 Is Associated with Higher Obesity and Insulin Resistance during Pregnancy in a Non-Diabetic White British Population. PLoS ONE.

[136] Yajnik C.S., Deshmukh U.S. (2012). Fetal programming: Maternal nutrition and role of one-carbon metabolism. Rev. Endocr. Metab. Disord..

[137] Rosenblatt D.S., Whitehead V.M. (1999). Cobalamin and folate deficiency: Acquired and hereditary disorders in children. Semin. Hematol..

[138] Evatt M.L., Terry P.D., Ziegler T.R., Oakley G.P. (2010). Association between vitamin B12-containing supplement consumption and prevalence of biochemically defined B12 deficiency in adults in NHANES III (third national health and nutrition examination survey). Public Health Nutr..

[139] USDA National Nutrient Database for Standard Reference. https://ndb.nal.usda.gov.

[140] Banca Dati Di Composizione Degli Alimenti per Gli Studi Epidemiologici in Italia (BDA). http://www.bda-ieo.it.

[141] Doscherholmen A., McMahon J., Ripley D. (1975). Vitamin B12 absorption from eggs. Proc. Soc. Exp. Biol. Med..

[142] Dubey R.C., Maheshwari D.K. (2013). A Textbook of Microbiology.

[143] Allen L., de Benoist B., Dary O., Hurrell R. (2006). Guidelines on Food Fortification with Micronutrients.

[144] Green R. (2009). Is it time for vitamin B-12 fortification? What are the questions?. Am. J. Clin. Nutr..

[145] Allen L.H., Rosenberg I.H., Oakley G.P., Omenn G.S. (2010). Considering the case for vitamin B12 fortification of flour. Food Nutr. Bull..

[146] Stover P.J. (2004). Physiology of folate and vitamin B12 in health and disease. Nutr. Rev..

[147] Bhuiyan A., Dash S., Shahriar S., Nahid F., Arefin S. (2011). A case of sub-acute combined degeneration of the spinal cord with associated pernicious anaemia. Pulse.

[148] Tan L.T.H., Ho K.K.F., Fong G.C.Y., Ong K.L. (2010). Subacute combined degeneration of the spinal cord. Hong Kong J. Emerg. Med..

[149] EFSA Panel on Dietetic Products, Nutrition, and Allergies (NDA) (2016). Tolerable Upper Intake Levels for Vitamins and Minerals.

[150] Shibuya K., Misawa S., Nasu S., Sekiguchi Y., Beppu M., Iwai Y., Mitsuma S., Isose S., Arimura K., Kaji R. (2014). Safety and efficacy of intravenous ultra-high dose methylcobalamin treatment for peripheral neuropathy: A phase I/II open label clinical trial. Intern. Med..

[151] Carmel R. (2008). How I treat cobalamin (vitamin B12) deficiency. Blood.

[152] Sharabi A., Cohen E., Sulkes J., Garty M. (2003). Replacement therapy for vitamin B12 deficiency: Comparison between the sublingual and oral route. Br. J. Clin. Pharmacol..

[153] Van Walraven C., Austin P., Naylor C.D. (2001). Vitamin B12 injections versus oral supplements. How much money could be saved by switching from injections to pills?. Can. Fam. Physician.

[154] Thakkar K., Billa G. (2015). Treatment of vitamin B12 deficiency-methylcobalamine? Cyancobalamine? Hydroxocobalamin?-clearing the confusion. Eur. J. Clin. Nutr..

[155] Zhang Y.F., Ning G. (2008). Mecobalamin. Expert Opin. Investig. Drugs.

[156] Institute of Medicine (2005). Dietary Reference Intakes for Energy, Carbohydrate, Fiber, Fat, Fatty Acids, Cholesterol, Protein, and Amino Acids (Macronutrients).

[157] Doi K., Matsuura M., Kawara A., Tanaka T., Baba S. (1983). Influence of dietary fiber (konjac mannan) on absorption of vitamin B12 and vitamin E. Tohoku J. Exp. Med..

[158] Alexander D., Ball M.J., Mann J. (1994). Nutrient intake and haematological status of vegetarians and age-sex matched omnivores. Eur. J. Clin. Nutr..

[159] Herbert V. (1992). Everyone should be tested for iron disorders. J. Am. Diet. Assoc..

[160] Appleby P., Roddam A., Allen N., Key T. (2007). Comparative fracture risk in vegetarians and nonvegetarians in EPIC-Oxford. Eur. J. Clin. Nutr..

[161] Herzlich B., Herbert V. (1984). The role of the pancreas in cobalamin (vitamin B12) absorption. Am. J. Gastroenterol..

[162] Bauman W.A., Shaw S., Jayatilleke E., Spungen A.M., Herbert V. (2000). Increased intake of calcium reverses vitamin B12 malabsorption induced by metformin. Diabetes Care.

[163] Li D., Yu X.M., Xie H.B., Zhang Y.H., Wang Q., Zhou X.Q., Yu P., Wang L.J. (2007). Platelet phospholipid n-3 PUFA negatively associated with plasma homocysteine in middle-aged and geriatric hyperlipaemia patients. Prostaglandins Leukot. Essent. Fat. Acids.

[164] Kulkarni A., Mehendale S., Pisal H., Kilari A., Dangat K., Salunkhe S., Taralekar V., Joshi S. (2011). Association of omega-3 fatty acids and homocysteine concentrations in pre-eclampsia. Clin. Nutr..

[165] Grundt H., Nilsen D.W., Mansoor M.A., Hetland Ø., Nordøy A. (2003). Reduction in homocysteine by n-3 polyunsaturated fatty acids after 1 year in a randomised double-blind study following an acute myocardial infarction: No effect on endothelial adhesion properties. Pathophysiol. Haemost. Thromb..

[166] Pooya S.H., Jalali M.D., Jazayery A.D., Saedisomeolia A., Eshraghian M.R., Toorang F. (2010). The efficacy of omega-3 fatty acid supplementation on plasma homocysteine and malondialdehyde levels of type 2 diabetic patients. Nutr. Metab. Cardiovasc. Dis..

[167] Zeman M., Zák A., Vecka M., Tvrzická E., Písaríková A., Stanková B. (2006). *N*-3 fatty acid supplementation decreases plasma homocysteine in diabetic dyslipidemia treated with statin-fibrate combination. J. Nutr. Biochem..

[168] Rosell M.S., Lloyd-Wright Z., Appleby P.N., Sanders T.A., Allen N.E., Key T.J. (2005). Long-chain *n*-3 polyunsaturated fatty acids in plasma in British meat-eating, vegetarian, and vegan men. Am. J. Clin. Nutr..

[169] Li D., Turner A., Sinclair A.J. (2002). Relationship between platelet phospholipid FA and mean platelet volume in healthy men. Lipids.

[170] Adams J.F. (1964). The urinary excretion and tissue retention of Cyanocobalamin by subjects given repeated parenteral doses. J. Clin. Pathol..

[171] Allen R.H., Stabler S.P. (2008). Identification and quantitation of cobalamin and cobalamin analogues in human feces. Am. J. Clin. Nutr..

[172] Kondo H., Binder M.J., Kolhouse J.F., Smythe W.R., Podell E.R., Allen R.H. (1982). Presence and formation of cobalamin analogues in multivitamin-mineral pills. J. Clin. Investig..

[173] Takenaka S., Sugiyama S., Watanabe F., Abe K., Tamura Y., Nakano Y. (1997). Effects of Carnosine and Anserine on the Destruction of Vitamin B12 with Vitamin C in the Presence of Copper. Biosci. Biotechnol. Biochem..

[174] Miyamoto E., Kittaka-Katsura H., Adachi S., Watanabe F. (2005). Assay of vitamin B12 in edible bamboo shoots. Vitamins.

[175] Kittaka-Katsura H., Watanabe F., Nakano Y. (2004). Occurrence of vitamin B12 in green, blue, red, and black tea leaves. J. Nutr. Sci. Vitaminol..

[176] La Guardia M., Venturella G., Venturella F. (2005). On the chemical composition and nutritional value of pleurotus taxa growing on umbelliferous plants (*Apiaceae*). J. Agric. Food Chem..

[177] Watanabe F., Schwarz J., Takenaka S., Miyamoto E., Ohishi N., Nelle E., Hochstrasser R., Yabuta Y. (2012). Characterization of vitamin B_12_compounds in the wild edible mushrooms black trumpet (*Craterellus cornucopioides*) and golden chanterelle (*Cantharellus cibarius*). J. Nutr. Sci. Vitaminol..

[178] Bito T., Teng F., Ohishi N., Takenaka S., Miyamoto E., Sakuno E., Terashima K., Yabuta Y., Watanabe F. (2014). Characterization of vitamin B12 compounds in the fruiting bodies of shiitake mushroom (*Lentinula edodes*) and bed logs after fruiting of the mushroom. Mycoscience.

[179] Watanabe F., Takenaka S., Katsura H., Masumder S.A., Abe K., Tamura Y., Nakano Y. (1999). Dried green and purple lavers (Nori) contain substantial amounts of biologically active vitamin B(12) but less of dietary iodine relative to other edible seaweeds. J. Agric. Food Chem..

[180] Miyamoto E., Yabuta Y., Kwak C.S., Enomoto T., Watanabe F. (2009). Characterization of vitamin B12 compounds from Korean purple laver (*Porphyra* sp.) products. J. Agric. Food Chem..

[181] Yamada S., Shibata Y., Takayama M., Narita Y., Sugawara K., Fukuda M. (1996). Content and characteristics of vitamin B12 in some seaweeds. J. Nutr. Sci. Vitaminol..

[182] Suzuki H. (1995). Serum vitamin B12 levels in young vegans who eat brown rice. J. Nutr. Sci. Vitaminol..

[183] Dagnelie P.C., van Staveren W.A., van den Berg H. (1991). Vitamin B-12 from algae appears not to be bioavailable. Am. J. Clin. Nutr..

[184] Watanabe F., Takenaka S., Kittaka-Katsura H., Ebara S., Miyamoto E. (2002). Characterization and bioavailability of vitamin B12-compounds from edible algae. J. Nutr. Sci. Vitaminol..

[185] Baroni L., Scoglio S., Benedetti S., Bonetto C., Pagliarani S., Benedetti Y., Rocchi M., Canestrari F. (2009). Effect of a Klamath algae product (“AFA-B12”) on blood levels of vitamin B12 and homocysteine in vegan subjects: A pilot study. Int. J. Vitam. Nutr. Res..

[186] Miyamoto E., Tanioka Y., Nakao T., Barla F., Inui H., Fujita T., Watanabe F., Nakano Y. (2006). Purification and characterization of a corrinoid-compound in an edible cyanobacterium *Aphanizomenon flos-aquae* as a nutritional supplementary food. J. Agric. Food Chem..

[187] Pulz O., Gross W. (2004). Valuable products from biotechnology of microalgae. Appl. Microbiol. Biotechnol..

[188] Merchant R.E., Phillips T.W., Udani J. (2015). Nutritional Supplementation with *Chlorella pyrenoidosa* Lowers Serum Methylmalonic Acid in Vegans and Vegetarians with a Suspected Vitamin B_12_ Deficiency. J. Med. Food.

[189] Kittaka-Katsura H., Fujita T., Watanabe F., Nakano Y. (2002). Purification and characterization of a corrinoid compound from *Chlorella* tablets as an algal health food. J. Agric. Food Chem..

[190] Watanabe F., Tanioka Y., Miyamoto E., Fujita T., Takenaka H., Nakano Y. (2007). Purification and characterization of corrinoid-compounds from the dried powder of an edible cyanobacterium, *Nostoc commune* (Ishikurage). J. Nutr. Sci. Vitaminol..

[191] Watanabe F., Katsura H., Takenaka S., Fujita T., Abe K., Tamura Y., Nakatsuka T., Nakano Y. (1999). Pseudovitamin B(12) is the predominant cobamide of an algal health food, spirulina tablets. J. Agric. Food Chem..

[192] Herbert V., Drivas G. (1982). Spirulina and vitamin B 12. JAMA.

[193] Tanioka Y., Yabuta Y., Miyamoto E., Inui H., Watanabe F. (2008). Analysis of vitamin B12 in food by silica gel 60 TLC and bioautography with vitamin B12-dependent *Escherichia coli* 215. J. Liq. Chromatogr. Relat. Technol..

[194] Denter J., Bisping B. (1994). Formation of B-vitamins by bacteria during the soaking process of soybeans for tempe fermentation. Int. J. Food Microbiol..

[195] Nout M.J.R., Rombouts F.M. (1990). Recent developments in tempe research. J. Appl. Bacteriol..

[196] Okada N., Hadioetomo P.S., Nikkuni S., Katoh K., Ohta T. (1983). Vitamin B12 content of fermented foods in the tropics. Rep. Natl. Food Res. Inst..

[197] Kwak C.S., Hwang J.Y., Watanabe F., Park S.C. (2008). Vitamin B12 contents in some Korean fermented foods and edible seaweeds. Korean Nutr..

[198] Babuchowski A., Laniewska-Moroz L., Warminska-Radyko I. (1999). Propionibacteria in fermented vegetables. Lait.

[199] Mozafar A. (1994). Enrichment of some B-vitamins in plants with application of organic fertilizers. Plant Soil.

[200] Sato K., Kudo Y., Muramatsu K. (2004). Incorporation of a high level of vitamin B12 into a vegetable, kaiware daikon (Japanese radish sprout), by the absorption from its seeds. Biochim. Biophys. Acta.

[201] Bito T., Ohishi N., Hatanaka Y., Takenaka S., Nishihara E., Yabuta Y., Watanabe F. (2013). Production and characterization of cyanocobalamin-enriched lettuce (*Lactuca sativa* L.) grown using hydroponics. J. Agric. Food Chem..

[202] Gu Q., Zhang C., Song D., Li P., Zhu X. (2015). Enhancing vitamin B12 content in soy-yogurt by *Lactobacillus reuteri*. Int. J. Food Microbiol..

[203] Rizzo G., Baroni L. (2016). Health and ecological implications of fish consumption: A deeper insight. Med. J. Nutr. Metab..

[204] Craig W.J. (2010). Nutrition concerns and health effects of vegetarian diets. Nutr. Clin. Pract..

[205] Clinical Trials NCT02679833. NCT02679833.

[206] Yazaki Y., Chow G., Mattie M. (2006). A single-center, double-blinded, randomized controlled study to evaluate the relative efficacy of sublingual and oral vitamin B-complex administration in reducing total serum homocysteine levels. J. Altern. Complement. Med..

[207] Vidal-Alaball J., Butler C.C., Cannings-John R., Goringe A., Hood K., McCaddon A., McDowell I., Papaioannou A. (2005). Oral vitamin B12 versus intramuscular vitamin B12 for vitamin B12 deficiency. Cochrane Database Syst. Rev..

[208] Oladipo O., Rosenblatt D.S., Watkins D., Miousse I.R., Sprietsma L., Dietzen D.J., Shinawi M. (2011). Cobalamin F disease detected by newborn screening and follow-up on a 14-year-old patient. Pediatrics.

[209] Waggoner D.J., Ueda K., Mantia C., Dowton S.B. (1998). Methylmalonic aciduria (*cblF*): Case report and response to therapy. Am. J. Med. Genet..

[210] Andersson H.C., Shapira E. (1998). Biochemical and clinical response to hydroxocobalamin versus cyanocobalamin treatment in patients with methylmalonic acidemia and homocystinuria (*cblC*). J. Pediatr..

[211] Linnell J.C., Smith A.D., Smith C.L., Wilson J., Matthews D.M. (1968). Effects of smoking on metabolism and excretion of vitamin B12. Br. Med. J..

[212] Shepherd G., Velez L.I. (2008). Role of hydroxocobalamin in acute cyanide poisoning. Ann. Pharmacother..

[213] Freeman A.G. (1992). Cyanocobalamin—A case for withdrawal: Discussion paper. J. R. Soc. Med..

[214] Freeman A.G. (1996). Hydroxocobalamin versus cyanocobalamin. J. R. Soc. Med..

[215] Balta I., Ozuguz P. (2014). Vitamin B12-induced acneiform eruption. Cutan. Ocul. Toxicol..

[216] Cianci A., Colacurci N., Paoletti A.M., Perino A., Cicinelli E., Maffei S., Di Martino M., Daguati R., Stomati M., Pilloni M. (2015). Soy isoflavones, inulin, calcium, and vitamin D3 in post-menopausal hot flushes: An observational study. Clin. Exp. Obstet. Gynecol..

[217] Cignini P., Mangiafico L., Padula F., D’Emidio L., Dugo N., Aloisi A., Giorlandino C., Vitale S.G. (2015). Supplementation with a dietary multicomponent (Lafergin^®^) based on Ferric Sodium EDTA (Ferrazone^®^): Results of an observational study. J. Prenat. Med..

[218] Colonese F., Laganà A.S., Colonese E., Sofo V., Salmeri F.M., Granese R., Triolo O. (2015). The pleiotropic effects of vitamin D in gynaecological and obstetric diseases: An overview on a hot topic. Biomed. Res. Int..

[219] Laganà A.S., Vitale S.G., Nigro A., Sofo V., Salmeri F.M., Rossetti P., Rapisarda A.M., La Vignera S., Condorelli R.A., Rizzo G. (2016). Pleiotropic Actions of Peroxisome Proliferator-Activated Receptors (PPARs) in Dysregulated Metabolic Homeostasis, Inflammation and Cancer: Current Evidence and Future Perspectives. Int. J. Mol. Sci..

[220] Misra U.K., Kalita J., Singh S.K., Rahi S.K. (2016). Oxidative stress markers in vitamin B12 deficiency. Mol. Neurobiol..

